# Identification of Key Genes and Prognostic Value Analysis in Hepatocellular Carcinoma by Integrated Bioinformatics Analysis

**DOI:** 10.1155/2019/3518378

**Published:** 2019-11-22

**Authors:** Meng Wang, Licheng Wang, Shusheng Wu, Dongsheng Zhou, Xianming Wang

**Affiliations:** ^1^General Surgery Department, Ward 3, Central Hospital of Zi Bo, Shandong 255000, China; ^2^Department of Urology, Tongji Hospital, Tongji University School of Medicine, Shanghai 200065, China; ^3^Department of General Surgery, Shandong Provincial Qianfoshan Hospital, Shandong University, Shandong 250014, China; ^4^Department of General Surgery, The First Affiliated Hospital of Shandong First Medical University, Shandong 250014, China

## Abstract

Emerging evidence indicates that various functional genes with altered expression are involved in the tumor progression of human cancers. This study is aimed at identifying novel key genes that may be used for hepatocellular carcinoma (HCC) diagnosis, prognosis, and targeted therapy. This study included 3 expression profiles (GSE45267, GSE74656, and GSE84402), which were obtained from the Gene Expression Omnibus (GEO). GEO2R was used to analyze the differentially expressed genes (DEGs) between HCC and normal samples. The functional and pathway enrichment analysis was performed by the Database for Annotation, Visualization and Integrated Discovery. A protein-protein interaction (PPI) network of the identified DEGs was constructed using the Search Tool for the Retrieval of Interacting Gene, and hub genes were identified. ONCOMINE and CCLE databases were used to verify the expression of the hub genes in HCC tissues and cells. Kaplan-Meier plotter was used to assess the effects of the hub genes on the overall survival of HCC patients. A total of 99 DEGs were identified from the 3 expression profiles. These DEGs were enriched with functional processes and pathways related to HCC pathogenesis. From the PPI network, 5 hub genes were identified. The expression of the 5 hub genes was all upregulated in HCC tissues and cells compared with the control tissues and cells. Kaplan-Meier survival curves indicated that high expression of cyclin-dependent kinase (CDK1), cyclin B1 (CCNB1), cyclin B2 (CCNB2), MAD2 mitotic arrest deficient-like 1 (MAD2L1), and topoisomerase II*α* (TOP2A) predicted poor overall survival in HCC patients (all log-rank *P* < 0.01). These results revealed that the DEGs may serve as candidate key genes during HCC pathogenesis. The 5 hub genes, including CDK1, CCNB1, CCNB2, MAD2L1, and TOP2A, may serve as promising prognostic biomarkers in HCC.

## 1. Introduction

Hepatocellular carcinoma (HCC) remains a serious health burden and is the second leading cause of cancer mortality worldwide [1]. Statistical data have indicated that the morbidity and mortality of HCC have been increasing in recent years, mainly due to the increased infection of hepatitis C virus [2]. Researchers have identified several established risk factors for the occurrence of HCC, such as liver cirrhosis, viral infection, metabolic disorder, and heavy alcohol consumption [3]. Despite advances in various therapeutic strategies, such as surgery, chemotherapy, radiotherapy, and biologics, the prognosis and outcomes remain poor in patients suffering from HCC [4]. Therefore, efficient diagnosis and prognosis remain great challenges for HCC treatment.

It is generally considered that tumorigenesis is a complex process with a wide spectrum of genetic alterations [5]. These genes typically exhibit aberrant expression patterns and have clinical significance in cancer diagnosis and prognosis [6]. Currently, some molecules have been recognized as diagnostic and prognostic biomarkers in HCC. For example, the high expression of peroxiredoxin 1 (Prdx1) is associated with tumor development and overall survival of HCC patients and serves as a candidate biomarker for the screening and prediction of this malignancy [7]. Sulfite oxidase (SUOX) expression is downregulated during tumorigenesis of HCC and is correlated with HCC diagnosis and prognosis [8]. Upregulated expression of distal-less homeobox gene 4 (DLX4) in HCC samples has been shown to be associated with poor prognosis of HCC patients [9]. Similarly, the altered alpha-fucosidase (AFU) expression has significant prognostic value in HCC patients and acts as a potential target for HCC-targeted therapy [10]. However, the available biomarkers are not suitable for all the HCC cases due to the limitations of sensitivity and specificity. Accordingly, the identification of novel functional genes may contribute to the understanding of tumor pathogenesis and the improvement of diagnosis and prognosis of HCC.

In recent research, differentially expressed genes (DEGs) in tumor samples compared with normal samples can be identified using gene expression profiling arrays [11, 12]. Some key molecules have also been reported in HCC using bioinformatics analysis [13, 14]. However, the number of the identified functional genes is far from sufficient to explain the mechanisms underlying the pathogenesis of HCC. Thus, this study used bioinformatics analyses to further identify key genes in HCC progression from 3 gene expression profiles from the Gene Expression Omnibus (GEO) database and assessed the clinical significance of the DEGs in HCC prognosis. The expression and prognostic value of the identified key genes were further verified using the data from The Cancer Genome Atlas (TCGA) database.

## 2. Materials and Methods

### 2.1. Data Collection

In this study, we firstly downloaded 3 gene expression profiles from GEO database (http://www.ncbi.nlm.nih.gov/geo), including GSE45267, GSE74656, and GSE84402. The inclusion criteria for the expression profiles were as follows: (1) the samples detected are tissues, (2) all tissues are diagnosed with HCC tissues and normal tissues, (3) gene expression profiling of mRNA, (4) samples collected from the same racial population, (4) probes can be converted into the corresponding gene symbols, and (5) complete information for our analyses. The array data of GSE45267 included 49 HCC tumor tissues and 38 normal tissues. GSE74656 contained 10 samples, including 5 HCC tumors and 5 adjacent normal tissues. GSE84402 was comprised of 14 tumor tissues and 14 adjacent noncancerous tissues [15].

### 2.2. Data Processing

The DEGs between the HCC samples and normal samples were analyzed by GEO2R (http://www.ncbi.nlm.nih.gov/geo/geo2r), which is a built-in online tool of GEO [16]. Adjusted *P* value and |log fold change| (|log FC|) were used to evaluate the significance of DEGs, and adjusted *P* < 0.05 and ∣log FC∣ > 2 were set as the cutoff criteria.

### 2.3. Functional and Pathway Enrichment Analysis

The Database for Annotation, Visualization and Integrated Discovery (DAVID, http://david.ncifcrf.gov/) is an essential program for the comprehensive gene function analysis, which aids the researchers to understand the biological significance of abundant genes [17]. Gene ontology (GO) analysis and Kyoto Encyclopedia of Genes and Genome (KEGG) pathway enrichment analysis were performed for the obtained DEGs. A result with a *P* < 0.05 was considered statistically significant.

### 2.4. PPI Network Construction and Module Selection

Since the interactions between proteins represent the pivotal events during cellular biological processes, we constructed a protein-protein interaction (PPI) network of the identified DEGs using the Search Tool for the Retrieval of Interacting Gene (STRING, http://string.embl.de/) database [18]. The PPI network was visualized using Cytoscape (version 3.7.0) [19], and a confidence score ≥ 0.7 was used as the cutoff criterion. Subsequently, the modules of the PPI network were screened by the Molecular Complex Detection (MCODE) with the following parameters: degree cutoff = 2, node score cutoff = 0.2, *k*‐core = 2, and maximum depth = 100 [20].

### 2.5. Expression Analysis

The mRNA expression levels of the hub genes between HCC tissues and normal controls were analyzed using the ONCOMINE (http://www.oncomine.org) database [21], and the data were collected from three literatures [22–24]. In addition, the expression results were further confirmed in HCC cells by the CCLE (http://portals.broadinstitute.org/ccle/home) database [25]. The differences between two groups were analyzed using Student's *t*-test. *P* < 0.05 and fold changes > 2 were set as the cutoff criteria.

### 2.6. Survival Analysis of DEGs

The prognostic value of the identified hub genes in HCC was further assessed by the Kaplan-Meier plotter (KM plotter, http://www.kmplot.com/analysis/) [26]. The analysis included 364 patients, and their KM survival curves were conducted. In addition, the KM survival curves for patients with different tumor stages were separately plotted. However, only 5 patients were diagnosed with tumor stage 4, and the curve could not be performed due to the limited sample size. The actual number in the other stages can be lower due to missing expression values and/or incomplete survival data. The gene expression was grouped using a cutoff value that is located between the lower and upper quartiles and computed by the Kaplan-Meier plotter with a best performing threshold. The log-rank *P* value for the different survival distribution between the low and high expression group was assessed, and the hazard radio (HR) with 95% confidence interval (95% CI) was calculated and plotted on the webpage.

### 2.7. Verification of Expression and Prognostic Value of DEGs Using TCGA Data

To confirm the clinical significance of the 5 hub genes in the prognosis of HCC, data from TCGA database were further assessed using the Gene Expression Profiling Interactive Analysis (GEPIA), which is a web-based tool to deliver fast and customizable functionalities based on TCGA data [27]. The expression patterns in HCC tissues and the Kaplan-Meier survival curves were all performed using the GEPIA.

## 3. Results

### 3.1. Identification of DEGs in HCC

**Figure 1:**
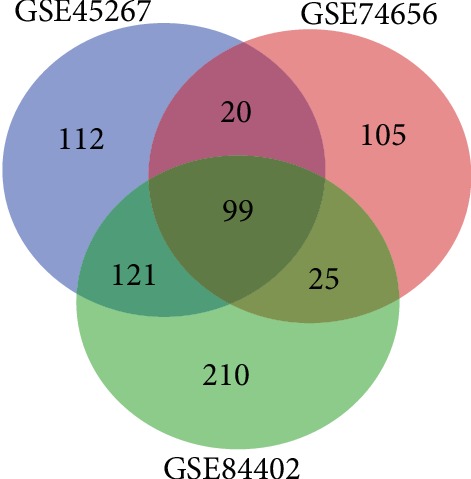
DEG identification in 3 mRNA expression profiles (GSE45267, GSE74656, and GSE84402). A total of 99 DEGs were identified from the 3 expression profiles. DEGs: differentially expressed genes.

According to the GEO2R analysis, a total of 352, 249, and 455 DEGs were, respectively, identified in GSE45267, GSE74656, and GSE84402. Among these DEGs, 99 genes with significant aberrant expression were extracted from all the three datasets (Figure 1), including 38 upregulated genes and 61 downregulated genes (Table 1).

### 3.2. GO Analysis and Pathway Enrichment Analysis of DEGs in HCC

The potential biological function of the identified 99 DEGs was assessed using GO analysis. As shown in Table 2, these genes were mainly enriched in biological processes related to cell division and mitotic nuclear division. Moreover, the potential signaling pathways which these DEGs involved were examined using KEGG analysis. From the results in Table 2, we found that the DEGs were mostly enriched in cell cycle and mineral absorption processes.

### 3.3. PPI Network Construction and Significant Module Analysis

**Figure 2:**
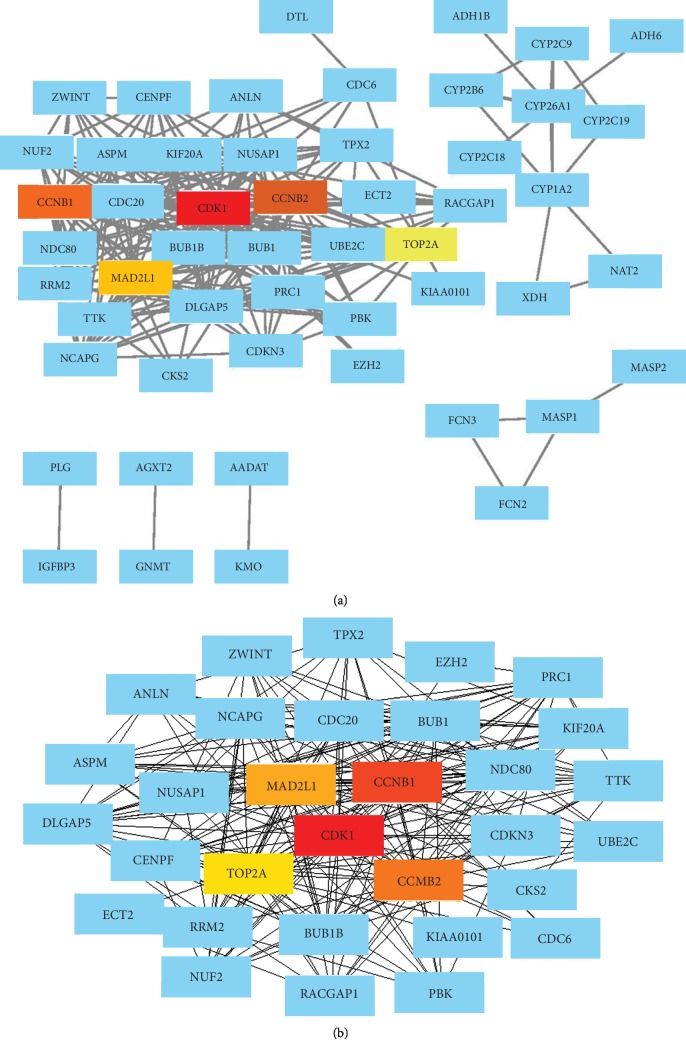
A PPI network of the DEGs and a significant module in the PPI network. (a) DEG PPI network contained 99 nodes and 298 edges. (b) The significant module obtained from the PPI network contained 32 nodes and 78 edges. The red, orange, and yellow nodes represented top 5 hub genes in the network.

The 99 identified DEGs were all filtered into the PPI network complex, which contained 99 nodes and 298 edges (Figure 2(a)). Furthermore, the most significant module was extracted from the PI network, containing 32 nodes and 78 edges (Figure 2(b)). In this module, 5 nodes with a degree > 10 were identified as hub genes, including cyclin-dependent kinase (CDK1), cyclin B1 (CCNB1), cyclin B2 (CCNB2), MAD2 mitotic arrest deficient-like 1 (MAD2L1), and topoisomerase II*α* (TOP2A) (Table 3).

### 3.4. CDK1 Expression Validation and Prognostic Value in HCC

**Figure 3:**
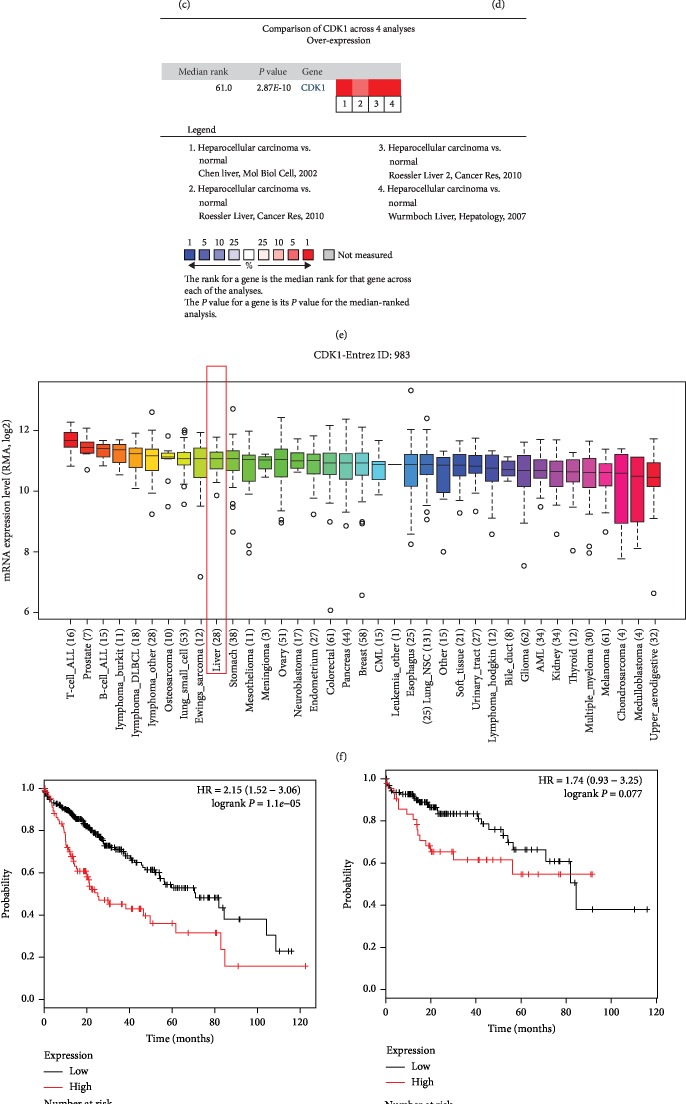
Expression and prognostic value of CDK1 in HCC. (a–e) The expression data collected from 4 datasets from ONCOMINE indicated that CDK1 expression was upregulated in HCC tissues compared with the normal control tissues and cells (all *P* < 0.05). (f) Expression of CDK1 was increased in HCC cells based on the data from CCLE. (g) The Kaplan-Meier survival curves revealed that the high CDK1 expression predicted worse overall survival compared with the low CDK1 expression in HCC patients (log-rank *P* < 0.001). (h) No significantly different survival times were observed between patients with high CDK1 expression and patients with low CDK1 expression at tumor stage 1 (log-rank *P* = 0.077). (i) High CDK1 expression predicted worse overall survival compared with the low CDK1 expression in HCC at tumor stage 2 (log-rank *P* = 0.0016). (j) High CDK1 expression predicted worse overall survival compared with the low CDK1 expression in HCC patients at tumor stage 3 (log-rank *P* = 0.013).

To further confirm the expression patterns of the 5 hub genes in HCC, we obtained 4 datasets from the ONCOMINE database to analyze the differential expression between HCC tissues and normal tissues. As shown in Figures 3(a)–3(d), the expression of CDK1 was significantly upregulated in HCC tissues compared with the normal controls in each dataset (all *P* < 0.05), and this difference was also statistically significant combined with the 4 datasets (*P* < 0.001, Figure 3(e)). Additionally, the mRNA expression of CDK1 in HCC cells was also analyzed using the CCLE database. The results shown in Figure 3(f) revealed that the CDK1 expression was also elevated in HCC cells. Furthermore, the Kaplan-Meier survival curves were constructed based on CDK1 expression in HCC patients. As shown in Figure 3(g), we considered that the high CDK1 expression was associated with poor overall survival compared with the low CDK1 expression in HCC patients (log-rank *P* < 0.001). In addition, survival curves for HCC patients with different tumor stages were also plotted, which showed that the high CDK1 predicts poor overall survival in patients with tumor stage 2 (log-rank *P* = 0.0016, Figure 3(i)) and tumor stage 3 (log-rank *P* = 0.013, Figure 3(j)). However, no significantly different survival times were observed between patients with high CDK1 expression levels and patients with low CDK1 expression levels at tumor stage 1 (log-rank *P* = 0.077, Figure 3(h)).

### 3.5. CCNB1 Expression Validation and Prognostic Value in HCC

**Figure 4:**
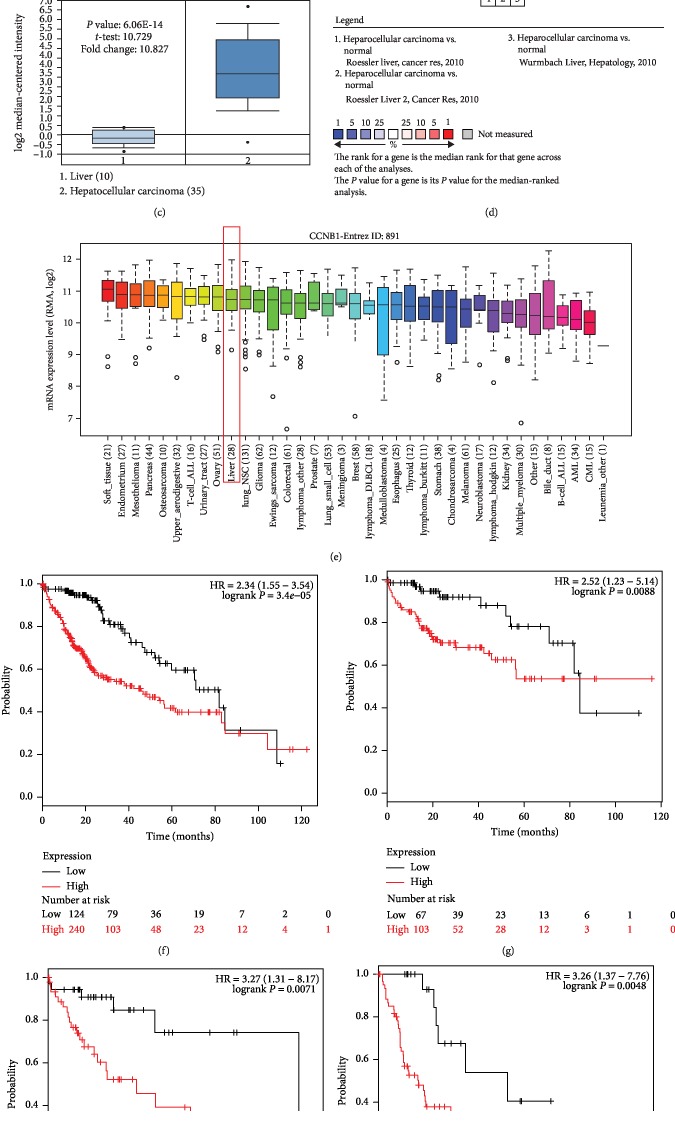
Expression and prognostic value of CCNB1 in HCC. (a–d) The expression data collected from 3 datasets from ONCOMINE indicated that CCNB1 expression was upregulated in HCC tissues compared with the normal controls (all *P* < 0.05). (e) Expression of CCNB1 was increased in HCC cells based on the data from CCLE. (f) The Kaplan-Meier survival curves revealed that the high CCNB1 expression predicted worse overall survival compared with the low CCNB1 expression in HCC patients (log-rank *P* < 0.001). (g) High CCNB1 expression predicted worse overall survival compared with the low CCNB1 expression in HCC patients at tumor stage 1 (log-rank *P* = 0.0088). (h) High CCNB1 expression predicted worse overall survival compared with the low CCNB1 expression in HCC patients at tumor stage 2 (log-rank *P* = 0.0071). (i) High CCNB1 expression predicted worse overall survival compared with the low CCNB1 expression in HCC patients at tumor stage 3 (log-rank *P* = 0.0048).

According to the expression investigation, we observed that the expression of CCNB1 was upregulated in both tissues and cells compared with the control tissues and cells (all *P* < 0.05, Figures 4(a)–4(e)). Furthermore, the prognostic value of CCNB1 was examined using the KM plotter. As shown in Figure 4(f), we considered that patients with the high CCNB1 expression level had poor overall survival compared with those with low CCNB1 expression level (log-rank *P* < 0.001). Moreover, the survival analysis for patients with different tumor stage revealed that the high CCNB1 expression level was associated with shorter survival time compared with the low CCNB1 expression level in HCC patients with tumor stage 1 (log-rank *P* = 0.0088, Figure 4(g)), tumor stage 2 (log-rank *P* = 0.0071, Figure 4(h)), and tumor stage 3 (log-rank *P* = 0.0048, Figure 4(i)).

### 3.6. CCNB2 Expression Validation and Prognostic Value in HCC

**Figure 5:**
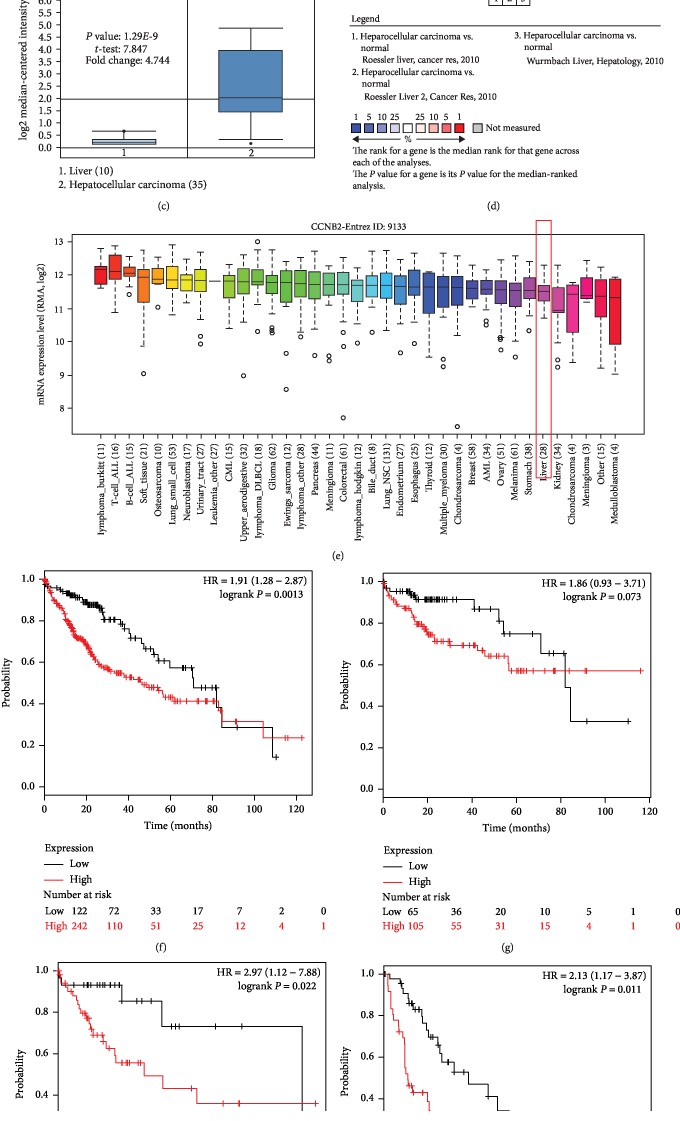
Expression and prognostic value of CCNB2 in HCC. (a–d) The expression data collected from 3 datasets from ONCOMINE indicated that CCNB2 expression was upregulated in HCC tissues compared with the normal controls (all *P* < 0.05). (e) Expression of CCNB2 was increased in HCC cells based on the data from CCLE. (f) The Kaplan-Meier survival curves revealed that the high CCNB2 expression predicted worse overall survival compared with the low CCNB2 expression in HCC patients (log-rank *P* = 0.0013). (g) No significantly different survival times were observed between patients with high CDK1 expression and patients with low CDK1 expression at tumor stage 1 (log-rank *P* = 0.073). (h) High CCNB2 expression predicted worse overall survival compared with the low CCNB2 expression in HCC patients at tumor stage 2 (log-rank *P* = 0.022). (i) High CCNB2 expression predicted worse overall survival compared with the low CCNB2 expression in HCC patients at tumor stage 3 (log-rank *P* = 0.011).

The expression patterns of CCNB2 in both HCC tissues and cells were greater than those in the normal control tissues and cells (all *P* < 0.05, Figures 5(a)–5(e)). To investigate the clinical significance of CCNB2 in HCC prognosis, the KM plotter was used to plot the survival curves for HCC patients. Patients with low CCNB2 expression had longer survival times compared with those with high CCNB2 expression (log-rank *P* = 0.0013, Figure 5(f)). Additionally, the effect of CCNB2 on overall survival of patients with different tumor stages were also assessed. The curves indicated that the high CCNB2 expression was associated with shorter survival times compared with the low CCNB2 expression in HCC patients with tumor stage 2 (log-rank *P* = 0.022, Figure 5(h)) and tumor stage 3 (log-rank *P* = 0.011, Figure 5(i)). However, no significantly different survival times were observed between patients with high CCNB2 expression levels and patients with low CCNB2 expression levels at tumor stage 1 (log-rank *P* = 0.073, Figure 5(g)).

### 3.7. MAD2L1 Expression Validation and Prognostic Value in HCC

**Figure 6:**
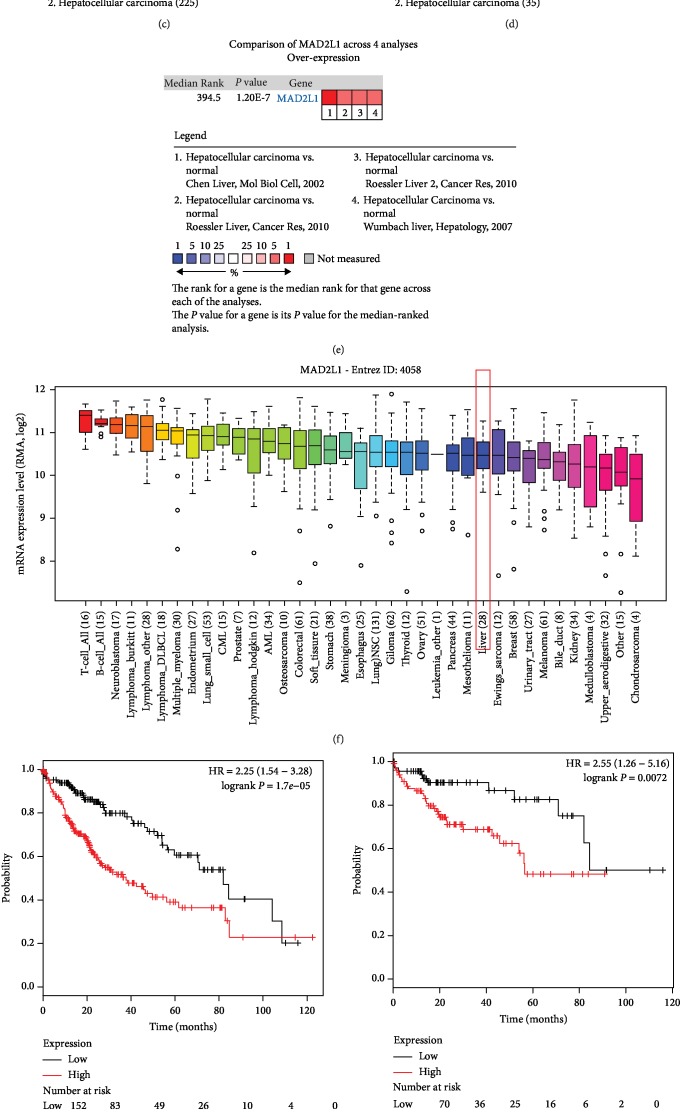
Expression and prognostic value of MAD2L1 in HCC. (a–e) The expression data collected from 4 datasets from ONCOMINE indicated that MAD2L1 expression was upregulated in HCC tissues compared with the normal controls (all *P* < 0.05). (f) Expression of MAD2L1 was increased in HCC cells based on the data from CCLE. (g) The Kaplan-Meier survival curves revealed that the high MAD2L1 expression predicted worse overall survival compared with the low MAD2L1 expression in HCC patients (log-rank *P* < 0.001). (h) High MAD2L1 expression predicted worse overall survival compared with the low MAD2L1 expression in patients with HCC at tumor stage 1 (log-rank *P* = 0.0072). (i) High MAD2L1 expression predicted worse overall survival compared with the low MAD2L1 expression in HCC patients at tumor stage 2 (log-rank *P* = 0.022). (j) High MAD2L1 expression predicted worse overall survival compared with the low MAD2L1 expression in patients with HCC at tumor stage 3 (log-rank *P* = 0.0015).

The expression of MAD2L1 was analyzed using the ONCOMINE database and the CCLE database and was proved to be upregulated in HCC tissues and cells compared with the control tissues and cells (all *P* < 0.05, Figures 6(a)–6(f)). Furthermore, the KM plotter was used to plot survival curves based on MAD2L1 expression in HCC patients. As shown in Figure 6(g), HCC patients with high MAD2L1 expression had poor overall survival compared with those with low MAD2L1 expression (log-rank *P* < 0.001). To explore the effect of MAD2L1 expression on HCC tumors with different tumor stages, survival analysis was performed for patients with tumor stages 1-3. The results indicated that the high MAD2L1 expression predicted shorter survival times compared with the low MAD2L1 expression in HCC patients with tumor stage 1 (log-rank *P* = 0.0072, Figure 6(h)), tumor stage 2 (log-rank *P* = 0.022, Figure 6(i)), and tumor stage 3 (log-rank *P* = 0.0015, Figure 6(j)).

### 3.8. TOP2A Expression Validation and Prognostic Value in HCC

**Figure 7:**
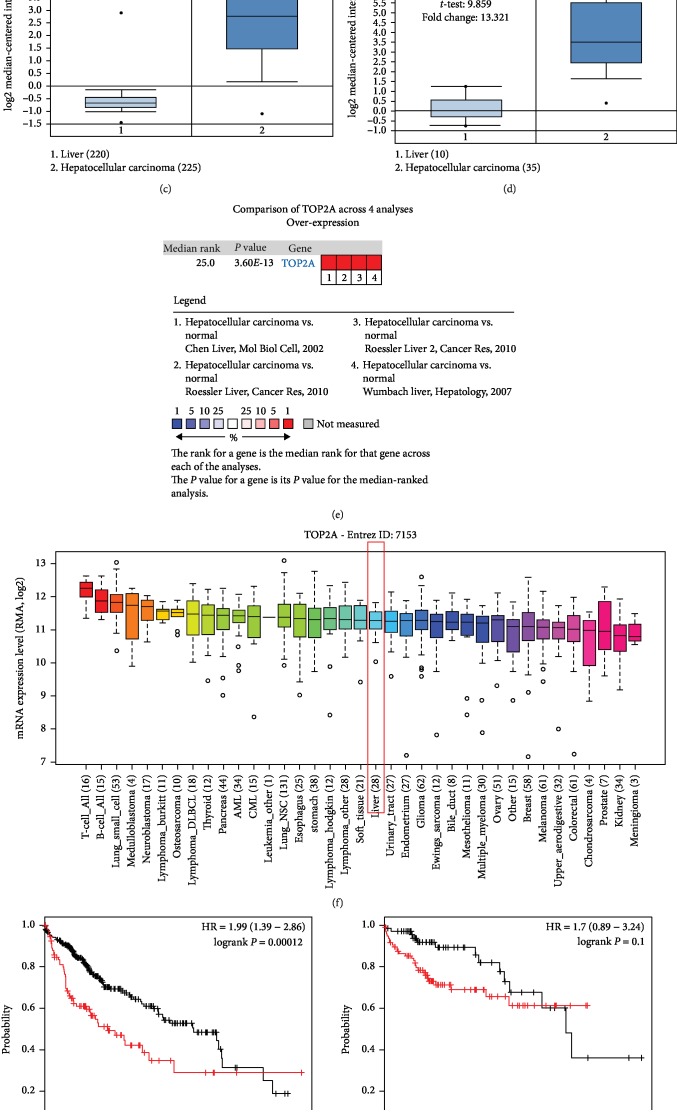
Expression and prognostic value of TOP2A in HCC. (a–e) The expression data collected from 4 datasets from ONCOMINE indicated that TOP2A expression was upregulated in HCC tissues compared with the normal controls (all *P* < 0.05). (f) Expression of TOP2A was increased in HCC cells based on the data from CCLE. (g) The Kaplan-Meier survival curves revealed that the high TOP2A expression predicted worse overall survival compared with the low TOP2A expression in HCC patients (log-rank *P* = 0.00012). (h) No significantly different survival times were found between patients with high TOP2A and patients with low TOP2A at tumor stage 1 (log-rank *P* = 0.1). (i) High TOP2A expression predicted worse overall survival compared with the low TOP2A expression in HCC patients at tumor stage 2 (log-rank *P* = 0.0073). (j) High TOP2A expression predicted worse overall survival compared with the low TOP2A expression in patients with HCC at tumor stage 3 (log-rank *P* = 0.00066).

The expression of TOP2A was investigated using the ONCOMINE database and the CCLE database, and the expression level was observed to be elevated in both HCC tissues and cells compared with the normal control tissues and cells (all *P* < 0.05, Figures 7(a)–7(f)). The survival analysis indicated that HCC patients with high TOP2A expression had poor overall survival compared with those with low TOP2A expression (log-rank *P* = 0.00012, Figure 7(g)). Moreover, we also found that the high TOP2A expression predicted poor overall survival compared with the low TOP2A expression in HCC patients with tumor stage 2 (log-rank *P* = 0.0073, Figure 7(i)) and tumor stage 3 (log-rank *P* = 0.00066, Figure 7(j)). In HCC patients with tumor stage 1, we observed that the trend of the high TOP2A expression was related to a shorter survival time, but the difference was not statistically significant (log-rank *P* = 0.1, Figure 7(h)).

### 3.9. Expression and Prognostic Value Verification Using TCGA Data

**Figure 8:**
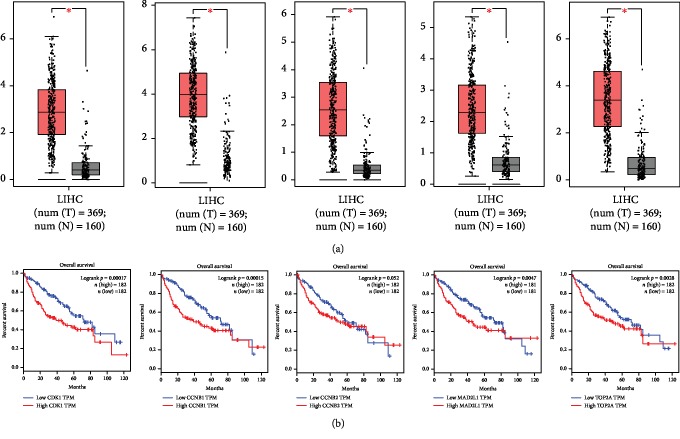
Expression and prognostic value verification using TCGA data. (a) TCGA data indicated that the expression levels of CDK1, CCNB1, CCNB2, MAD2L1, and TOP2A were all increased in tumor samples compared with normal controls (all *P* < 0.05; T: tumor; N: normal). (b) The patients with high CDK1, CCNB1, CCNB2, MAD2L1, and TOP2A expression had poor overall survival compared with those with low expression of these genes (log-rank *P* < 0.05 for CDK1, CCNB1, MAD2L1, and TOP2A; log-rank *P* = 0.052 for CCNB2).

By using the GEPIA, the expression patterns and prognostic value of the 5 hub genes were verified based on the data from TCGA database. Consistent with the expression results analyzed by ONCOMINE, the expression levels of CDK1, CCNB1, CCNB2, MAD2L1, and TOP2A assessed by TCGA data were all upregulated in tumor tissues compared with the normal controls (all *P* < 0.05, Figure 8(a)). Furthermore, the survival curves shown in Figure 8(b) indicated that high CDK1, CCNB1, MAD2L1, and TOP2A expression predicted poor overall survival (all log-rank *P* < 0.05). Although high expression of CCNB2 was also associated with shorter survival time, the difference of survival distribution between high and low expression groups was not statistically significant (log-rank *P* = 0.052).

## 4. Discussion

Accurate diagnosis and prognosis remain the great challenges for the improvement of HCC outcomes. To meet the clinical requirements of HCC treatment, various therapeutic methods have been developed in recent decades [28]. Moreover, targeted therapy, which is mainly dependent on genes that have pivotal roles during tumor pathogenesis, has attracted increasing attention [29]. These key genes are involved in tumor progression and typically have considerable clinical significance in the diagnosis and prognosis of various human cancers, including HCC. Ba and colleagues have reported that the serum expression of Golgi protein-73 (GP73) was higher in HCC patients compared with healthy individuals and serves as a novel biomarker for HCC diagnosis [30]. Similarly, upregulated expression of lysine specific demethylase 1 (LSD1) has been proven to be associated with poor prognosis of HCC [31]. To identify novel key genes that might be involved in HCC pathogenesis, we performed a systematic analysis of 3 expression profiles from GEO database using bioinformatics analysis. The DEGs in the expression profiles and the prognostic value of the key genes were assessed in the present study.

A total of 68 HCC samples and 57 normal control samples were included in the 3 expression profiles, and 99 DEGs were screened for further analyses. According to the functional and pathway enrichment analysis, the identified DEGs were shown to be enriched in biological processes that related to cell division and mitotic nuclear division and in signaling pathways that associated with cell cycle and mineral absorption. It is generally considered that cell division, mitotic nuclear division, and cell cycle are important cell processes in both normal and tumor cells [32]. Tumor-related key genes are typically involved in tumor progression by the regulation of these cell processes [33, 34].

Two interesting results were presented in our study. Firstly, the 99 DEGs included 6 members of the cytochrome P450 proteins (CYPs), including CYP1A2, CYP26A1, CYP2B6, CYP2C9, CYP2C19, and CYP2C18. CYPs represent a large group of enzymes with critical roles in the molecular metabolism [35]. They act critical roles in the development of various human cancers, including HCC, and mediate the metabolism of most of the procarcinogens [36]. The members of CYPs in our study were found to be downregulated in HCC tissues, indicating that the CYPs were involved in the progression of HCC and might inhibit the drug sensitivity. Secondly, the DEGs were found to be enriched in the mineral absorption pathway in this study. A previous research has demonstrated that mineral supplementation could improve the status of essential trace elements in biological samples collected from patients with liver cirrhosis and cancer [37]. Therefore, we speculated that the mineral absorption pathway might have effects on the maintenance of HCC tumor microenvironment, which needs to be analyzed and confirmed in future studies.

Furthermore, the PPI network of the DEGs was constructed, and 5 hub genes were extracted from a significant module, including CDK1, CCNB1, CCNB2, MAD2L1, and TOP2A. A study by Xing et al. [38] also focused on the DEGs in HCC tissues compared with the normal controls, and a same expression profile GSE45267 was analyzed and CCNB2 and TOP2A were identified as two of the hub genes, which was consistent with our corresponding data. Furthermore, the expression patterns of the 5 hub genes were found all upregulated in HCC tissues and cells compared with the normal controls, and their prognostic value was evaluated by plotting the Kaplan-Meier survival curves. The analysis results indicated that the expression levels of CDK1, CCNB1, CCNB2, MAD2L1, and TOP2A were associated with the overall survival of HCC patients. Additionally, the relationships between the hub genes and overall survival of HCC cases at different tumor stages were also observed, suggesting that these genes might serve as promising prognostic biomarkers in HCC.

CDK1 belongs to a serine/threonine kinase family and serves as a critical cell cycle-regulating protein. It has been widely investigated in human malignancies and has been found to be involved in tumor progression. In epithelial ovarian cancer, upregulated expression of CDK1 has been observed in cancer cells and promotes cancer growth and has a significant effect on the overall survival of patients [39]. In addition, a study scheduled by Luo et al. revealed that CDK1 had comprehensive effects on gene interaction networks in the tumor progression of cervical cancer and thus indicated the potential role of CDK1 as a therapeutic target [40]. In HCC, the aberrant CDK1 expression could regulate the apoptin-induced apoptosis with a pivotal role in tumor progression [41]. We also found the increased CDK1 expression in HCC samples and proved its prognostic value for cancer patients. The molecular mechanisms underlying the role of CDK1 in human cancers await more research.

CCNB1 and CCNB2 are two important cyclins that are closely correlated with the cell cycle and cell growth. Overexpression of CCNB1 and CCNB2 has been observed in some human cancer samples, and CCNB1 and CCNB2 possess clinical significance in the diagnosis and prognosis of various cancers, such as lung cancer [42] and pancreatic cancer [43]. Our study also showed the upregulated expression of CCNB1 and CCNB2 in both HCC tissues and cells and reported their prognostic value for the patients. However, the clinical significance verification using TCGA data showed that the difference between the survival distributes of low and high CCNB2 expression groups was not statistically significant, which might be due to the limited sample size and the incomplete survival information. Thus, although CCNB1 and CCNB2 have been previously reported to act as therapeutic target genes in HCC [44, 45], the clinical significance of CCNB1 and CCNB2 needs to be investigated in cancer-related research.

MAD2L1 plays an important role in spindle checkpoints during mitosis. Dysregulation of MAD2L1 induces the instability of chromosomes and chromosomal aneuploidy, which are common events in cancer [46]. It has been determined as a useful prognostic biomarker in some cancers, such as breast cancer [47] and lung adenocarcinoma [48]. An increased expression level of MAD2L1 was observed in HCC samples in the present study, which was consistent with the results from a study by Li et al. [49], which also found the overexpression of MAD2L1 in HCC. Collectively, the role of MAD2L1 in cancer pathogenesis and the related molecular mechanisms need to be assessed with in-depth studies.

TOP2A is an enzyme that is closely correlated with DNA replication, recombination, transcription, and chromatin remodeling [50]. The functional and clinical roles of TOP2A have been demonstrated in human cancers, including prostate cancer [51], breast cancer [52], and nasopharyngeal carcinoma [53]. This study showed elevated TOP2A expression levels in HCC tissues and cells and demonstrated its potential as a prognostic biomarker in this malignancy. The upregulation of TOP2A and its prognostic value have been reported in HCC in previous studies, which also revealed its correlation with tumor onset and chemoresistance [54]. Further studies should be carried out to explore the mechanisms underlying the role of TOP2A during cancer pathogenesis.

## 5. Conclusion

In conclusion, this study identified 99 DEGs from 3 expression profiles by integrated bioinformatics analysis. These DEGs may contain key genes involved in HCC pathogenesis. In addition, CDK1, CCNB1, CCNB2, MAD2L1, and TOP2A were the top five hub genes and serve as candidate prognostic biomarkers in HCC. The results of this study further enrich the number of key genes that may be involved in the pathogenesis of HCC and give in silico evidence for the key genes in the prognosis of HCC. However, our study fails to evaluate the clinical significance and biological function of the key genes in tumor samples by *in vitro* and *in vivo* analyses. Thus, further studies are needed to confirm the prognostic value and functional roles of these key genes in HCC.

## Figures and Tables

**Table 1:** Upregulated and downregulated DEGs.

DEGs	Gene name
Upregulated	ACSL4, ANLN, ASPM, BUB1, BUB1B, **CCNB1**, **CCNB2**, CDC20, CDC6, **CDK1**, CDKN3, CENPF, CKS2, CRNDE, CTHRC1, DLGAP5, DTL, ECT2, GINS1, GPC3, IGF2BP3, KIAA0101, KIF20A, **MAD2L1**, NCAPG, NDC80, NUF2, PBK, PEG10, PRC1, RACGAP1, RRM2, SULT1C2, **TOP2A**, TPX2, TTK, UBE2C, UBE2T
Downregulated	AADAT, ACSM3, ADH1B, ADH1C, AGXT2, AKR1D1, ALDH8A1, ALDOB, APOF, BCHE, CFHR3, CFHR4, CFP, CLEC1B, CLEC4G, CLEC4M, CLRN3, CNDP1, CRHBP, CXCL2, CYP1A2, CYP26A1, CYP2B6, CYP2C9, CYP2C19, CYP2C18, ESR1, FCN2, FCN3, GBA3, GNMT, GYS2, HAMP, HAO2, HGFAC, KCNN2, KLKB1, KMO, LCAT, MARCO, MASP2, MT1E, MT1F, MT1G, MT1H, MT1M, MT1X, NAT2, OIT3, PBLD, PCK1, PGLYRP2, PLG, SLC22A1, SLC25A47, STAB2, THRSP, TMEM27, TTC36, VIPR1, XDH

99 DEGs were identified from the three profile datasets, including 38 upregulated genes and 61 downregulated genes in the HCC tissues compared with the normal controls. The bold genes are hub genes. DEGs: differentially expressed genes.

**Table 2:** Functional and pathway enrichment analyses of DEGs in HCC.

Term	Description	*P* value	Count	Gene name
GO:0045926	Negative regulation of growth	9.99*E*-07	7	MT1M, GPC3, MT1E, MT1H, MT1X, MT1G, MT1F
GO:0051301	Cell division	1.30*E*-05	15	CDC6, **CDK1**, NUF2, TPX2, CENPF, NDC80, CDC20, UBE2C, **CCNB1**, **CCNB2**, **MAD2L1**, NCAPG, BUB1, CKS2, BUB1B
GO:0007067	Mitotic nuclear division	2.01*E*-05	13	**CDK1**, CDC6, **CCNB2**, NUF2, BUB1, TPX2, CENPF, BUB1B, NDC80, ANLN, CDC20, PBK, ASPM
GO:0071276	Cellular response to cadmium ion	4.32*E*-05	6	MT1E, CYP1A2, MT1H, MT1X, MT1G, MT1F
GO:0071294	Cellular response to zinc ion	8.05*E*-05	6	MT1M, MT1E, MT1H, MT1X, MT1G, MT1F
GO:0007094	Mitotic spindle assembly checkpoint	6.21*E*-03	5	**MAD2L1**, BUB1, CENPF, TTK, BUB1B
GO:0000922	Spindle pole	2.80*E*-02	7	**CCNB1**, CDC6, **MAD2L1**, PRC1, TPX2, CENPF, CDC20
GO:0007062	Sister chromatid cohesion	3.61*E*-02	7	**MAD2L1**, NUF2, BUB1, CENPF, BUB1B, NDC80, CDC20
KEGG:hsa04110	Cell cycle	4.15*E*-03	9	**CCNB1**, **CDK1**, CDC6, MAD2L1, **CCNB2**, BUB1, TTK, BUB1B, CDC20
KEGG:hsa04978	Mineral absorption	2.54*E*-02	5	MT1M, MT1E, MT1H, MT1X, MT1G, MT1F

DEGs: differentially expressed genes; GO: gene ontology; KEGG: Kyoto Encyclopedia of Genes and Genomes. The bold genes are hub genes.

**Table 3:** Top 5 hub genes in the PPI network.

Rank	Gene name	Score
1	CDK1	29
2	CCNB1	26
3	CCNB2	25
4	MAD2L1	24
5	TOP2A	23

PPI: protein-protein interaction.

## Data Availability

The data used to support the findings of this study are included within the article.

## References

[1] Torre L. A., Bray F., Siegel R. L., Ferlay J., Lortet-Tieulent J., Jemal A. (2015). Global cancer statistics, 2012. *CA: A Cancer Journal for Clinicians*.

[2] Njei B., Rotman Y., Ditah I., Lim J. K. (2015). Emerging trends in hepatocellular carcinoma incidence and mortality. *Hepatology*.

[3] White D. L., Kanwal F., El-Serag H. B. (2012). Association between nonalcoholic fatty liver disease and risk for hepatocellular cancer, based on systematic review. *Clinical Gastroenterology and Hepatology*.

[4] Ashhab A. A., Rodin H., Powell J., Debes J. D. (2017). Hepatocellular carcinoma diagnosis and surveillance: socioeconomic factors don’t seem to matter, unless you are an immigrant. *Journal of Hepatology*.

[5] Huang M. Y., Chen H. C., Yang I. P. (2013). Tumorigenesis and tumor progression related gene expression profiles in colorectal cancer. *Cancer Biomarkers*.

[6] Xie F. J., Lu H. Y., Zheng Q. Q. (2016). The clinical pathological characteristics and prognosis of FGFR1 gene amplification in non-small-cell lung cancer: a meta-analysis. *OncoTargets and Therapy*.

[7] Sun Q. K., Zhu J. Y., Wang W. (2014). Diagnostic and prognostic significance of peroxiredoxin 1 expression in human hepatocellular carcinoma. *Medical Oncology*.

[8] Jin G. Z., Yu W. L., Dong H. (2013). SUOX is a promising diagnostic and prognostic biomarker for hepatocellular carcinoma. *Journal of Hepatology*.

[9] Gao Y., Li Z., Guo X., Liu Y., Zhang K. (2014). DLX4 as a prognostic marker for hepatocellular carcinoma. *Neoplasma*.

[10] Wang K., Guo W., Li N. (2014). Alpha-1-fucosidase as a prognostic indicator for hepatocellular carcinoma following hepatectomy: a large-scale, long-term study. *British Journal of Cancer*.

[11] Sui W., Shi Z., Xue W. (2017). Circular RNA and gene expression profiles in gastric cancer based on microarray chip technology. *Oncology Reports*.

[12] Tantai J. C., Pan X. F., Zhao H. (2015). Network analysis of differentially expressed genes reveals key genes in small cell lung cancer. *European Review for Medical and Pharmacological Sciences*.

[13] Zhang C., Peng L., Zhang Y. (2017). The identification of key genes and pathways in hepatocellular carcinoma by bioinformatics analysis of high-throughput data. *Medical Oncology*.

[14] Mou T., Zhu D., Wei X. (2017). Identification and interaction analysis of key genes and microRNAs in hepatocellular carcinoma by bioinformatics analysis. *World Journal of Surgical Oncology*.

[15] Wang H., Huo X., Yang X. R. (2017). STAT3-mediated upregulation of lncRNA HOXD-AS1 as a ceRNA facilitates liver cancer metastasis by regulating SOX4. *Molecular Cancer*.

[16] Barrett T., Wilhite S. E., Ledoux P. (2013). NCBI GEO: archive for functional genomics data sets--update. *Nucleic Acids Research*.

[17] Huang D. W., Sherman B. T., Lempicki R. A. (2009). Systematic and integrative analysis of large gene lists using DAVID bioinformatics resources. *Nature Protocols*.

[18] Szklarczyk D., Franceschini A., Wyder S. (2015). STRING v10: protein-protein interaction networks, integrated over the tree of life. *Nucleic Acids Research*.

[19] Shannon P., Markiel A., Ozier O. (2003). Cytoscape: a software environment for integrated models of biomolecular interaction networks. *Genome Research*.

[20] Bader G. D., Hogue C. W. (2003). An automated method for finding molecular complexes in large protein interaction networks. *BMC Bioinformatics*.

[21] Rhodes D. R., Yu J., Shanker K. (2004). ONCOMINE: A Cancer Microarray Database and Integrated Data-Mining Platform. *Neoplasia*.

[22] Chen X., Cheung S. T., So S. (2002). Gene expression patterns in human liver cancers. *Molecular Biology of the Cell*.

[23] Roessler S., Jia H. L., Budhu A. (2010). A unique metastasis gene signature enables prediction of tumor relapse in early-stage hepatocellular carcinoma patients. *Cancer Research*.

[24] Wurmbach E., Chen Y. B., Khitrov G. (2007). Genome‐wide molecular profiles of HCV‐induced dysplasia and hepatocellular carcinoma. *Hepatology*.

[25] Petryszak R., Keays M., Tang Y. A. (2016). Expression Atlas update—an integrated database of gene and protein expression in humans, animals and plants. *Nucleic Acids Research*.

[26] Győrffy B., Surowiak P., Budczies J., Lánczky A. (2013). Online survival analysis software to assess the prognostic value of biomarkers using transcriptomic data in non-small-cell lung cancer. *PLoS One*.

[27] Tang Z., Li C., Kang B., Gao G., Li C., Zhang Z. (2017). GEPIA: a web server for cancer and normal gene expression profiling and interactive analyses. *Nucleic Acids Research*.

[28] Graf D., Vallböhmer D., Knoefel W. T. (2014). Multimodal treatment of hepatocellular carcinoma. *European Journal of Internal Medicine*.

[29] Fonseca A. L., da Silva V. L., da Fonsêca M. M. (2016). Bioinformatics analysis of the human surfaceome reveals new targets for a variety of tumor types. *International Journal of Genomics*.

[30] Ba M. C., Long H., Tang Y. Q., Cui S. Z. (2012). GP73 expression and its significance in the diagnosis of hepatocellular carcinoma: a review. *International Journal of Clinical and Experimental Pathology*.

[31] Zhao Z. K., Yu H. F., Wang D. R. (2012). Overexpression of lysine specific demethylase 1 predicts worse prognosis in primary hepatocellular carcinoma patients. *World Journal of Gastroenterology*.

[32] Hyun S. Y., Rosen E. M., Jang Y. J. (2012). Novel DNA damage checkpoint in mitosis: mitotic DNA damage induces re- replication without cell division in various cancer cells. *Biochemical and Biophysical Research Communications*.

[33] Kaseb H. O., Lewis D. W., Saunders W. S., Gollin S. M. (2016). Cell division patterns and chromosomal segregation defects in oral cancer stem cells. *Genes, Chromosomes & Cancer*.

[34] Wang X., Yu Q., Zhang Y., Ling Z., Yu P. (2015). Tectonic 1 accelerates gastric cancer cell proliferation and cell cycle progression in vitro. *Molecular Medicine Reports*.

[35] Korobkova E. A. (2015). Effect of natural polyphenols on CYP metabolism: implications for diseases. *Chemical Research in Toxicology*.

[36] Zhou J., Wen Q., Li S. F. (2016). Significant change of cytochrome P450s activities in patients with hepatocellular carcinoma. *Oncotarget*.

[37] Kazi T. G., Kolachi N. F., Afridi H. I. (2012). Effects of mineral supplementation on liver cirrhotic/cancer male patients. *Biological Trace Element Research*.

[38] Xing T., Yan T., Zhou Q. (2018). Identification of key candidate genes and pathways in hepatocellular carcinoma by integrated bioinformatical analysis. *Experimental and Therapeutic Medicine*.

[39] Yang W., Cho H., Shin H. Y. (2016). Accumulation of cytoplasmic Cdk1 is associated with cancer growth and survival rate in epithelial ovarian cancer. *Oncotarget*.

[40] Luo Y., Wu Y., Peng Y., Liu X., Bie J., Li S. (2016). Systematic analysis to identify a key role of CDK1 in mediating gene interaction networks in cervical cancer development. *Irish Journal of Medical Science*.

[41] Zhao J., Han S.-X., Ma J.-L. (2013). The role of CDK1 in apoptin-induced apoptosis in hepatocellular carcinoma cells. *Oncology Reports*.

[42] Qian X., Song X., He Y. (2015). CCNB2 overexpression is a poor prognostic biomarker in Chinese NSCLC patients. *Biomedicine & Pharmacotherapy*.

[43] Zhou L., Li J., Zhao Y. P. (2014). The prognostic value of Cyclin B1 in pancreatic cancer. *Medical Oncology*.

[44] Wang B., Xunsun, Liu J. Y. (2012). The effect of cell cycle and expression of cyclin B1 and cyclin C protein in hepatocellular carcinoma cell line HepG2 and SMMC-7721 after of silencing *β*-catenin gene. *Hepatogastroenterology*.

[45] Gao C. L., Wang G. W., Yang G. Q., Yang H., Zhuang L. (2018). Karyopherin subunit-*α* 2 expression accelerates cell cycle progression by upregulating CCNB2 and CDK1 in hepatocellular carcinoma. *Oncology Letters*.

[46] Li Y., Benezra R. (1996). Identification of a human mitotic checkpoint gene: hsMAD2. *Science*.

[47] Wang Z., Katsaros D., Shen Y. (2015). Biological and clinical significance of MAD2L1 and BUB1, genes frequently appearing in expression signatures for breast cancer prognosis. *PLoS One*.

[48] Shi Y. X., Zhu T., Zou T. (2016). Prognostic and predictive values of CDK1 and MAD2L1 in lung adenocarcinoma. *Oncotarget*.

[49] Li Y., Bai W., Zhang J. (2017). MiR-200c-5p suppresses proliferation and metastasis of human hepatocellular carcinoma (HCC) via suppressing MAD2L1. *Biomedicine & Pharmacotherapy*.

[50] Baldwin E. L., Osheroff N. (2005). Etoposide, topoisomerase II and cancer. *Current Medicinal Chemistry Anti-Cancer Agents*.

[51] de Resende M. F., Vieira S., Chinen L. T. D. (2013). Prognostication of prostate cancer based on TOP2A protein and gene assessment: TOP2A in prostate cancer. *Journal of Translational Medicine*.

[52] Moretti E., Desmedt C., Biagioni C. (2013). TOP2A protein by quantitative immunofluorescence as a predictor of response to epirubicin in the neoadjuvant treatment of breast cancer. *Future Oncology*.

[53] Lan J., Huang H. Y., Lee S. W. (2014). TOP2A overexpression as a poor prognostic factor in patients with nasopharyngeal carcinoma. *Tumour Biology*.

[54] Wong N., Yeo W., Wong W. L. (2009). TOP2A overexpression in hepatocellular carcinoma correlates with early age onset, shorter patients survival and chemoresistance. *International Journal of Cancer*.

